# Happy 70th birthday to Buddy Ratner!!

**DOI:** 10.1116/1.4985184

**Published:** 2017-06-12

**Authors:** Allan S. Hoffman

**Affiliations:** Emeritus Professor of Bioengineering, University of Washington, Seattle, Washington 98195

When I heard that Dave Castner was the Editor for a special issue of Biointer*phases* dedicated to celebrating Buddy's 70th birthday, I immediately offered to write a *short* article about Buddy Ratner's contributions to the biomaterials field. I did not think about it until now, in the spring of 2017, when the article has suddenly become due. And now faced with this deadline, I realize that it is *practically impossible* to write a “*short*” article about Buddy Ratner's contributions to the biomaterials field. Since he first arrived in Seattle in 1972 to join me as my postdoc, Buddy has contributed so many exciting, novel concepts to the biomaterials field, and he has published so many outstanding research papers in this field, that it is practically and literally impossible to summarize these contributions in one *short* article. However, I will try to do it.

Thus, in this short article, I will attempt to highlight just a few of the many exciting ideas that Buddy has brought to the biomaterials field over the past 45 years since he joined me as a postdoc in 1972.

## 1972 and beyond, in Seattle

To begin this journey, let me point out that Buddy did his Ph.D. thesis research on the *design and synthesis of hydrogels as new dialysis membranes*. He worked on these new and exciting materials at a relatively “specialized” university in Brooklyn, NY, known as “Brooklyn Polytechnic Institute.” Brooklyn Poly was famous because the top polymer scientists in the world taught and carried out their pioneering research on polymers there. Thus, Buddy had begun to work on *hydrogel biomaterials* before most contemporary scientists had ever heard the words “hydrogel” or “biomaterial.”

Buddy and I briefly met for the first time, when I lectured at Brooklyn Poly in 1969 on the research work that I was doing at Massachusetts Institute of Technology (MIT) on radiation-grafted hydrogels on surfaces. I had a grant from the U.S. Atomic Energy Commission (USAEC) to use ^60^Co radiation to graft poly(hydroxyethyl methacrylate) to hydrophobic biomaterial surfaces to make the surface “more hydrophilic, e.g., like the body” and to provide reactive –OH groups for attaching biomolecules such as heparin. In 1971, he wrote a letter to me (there was no e-mail in those days!) that he wanted to join me as a postdoc and to continue working on hydrogels in bio-related fields. I had just moved from MIT to the University of Washington (UW) in the fall of 1970, where I was just initiating a new program on medical applications of hydrogels. In 1971, I offered Buddy a postdoctoral position, and he accepted. He arrived in Seattle in early 1972 to begin his postdoc, and luckily (for me!) my grant from the USAEC provided the funding for him. Tom Horbett had joined me as my first postdoc in 1970, and so I was lucky to start in 1972 with a great research group that included Tom and Buddy. They became close colleagues and worked well together. They also became good friends as they both rose in rank to full, tenured Professors in our UW Bioengineering Department.

I can list the following among the many exciting new ideas and innovations in biomaterials that Buddy helped to create at UW.

## 1970s–1980s

**Fig. 1.**
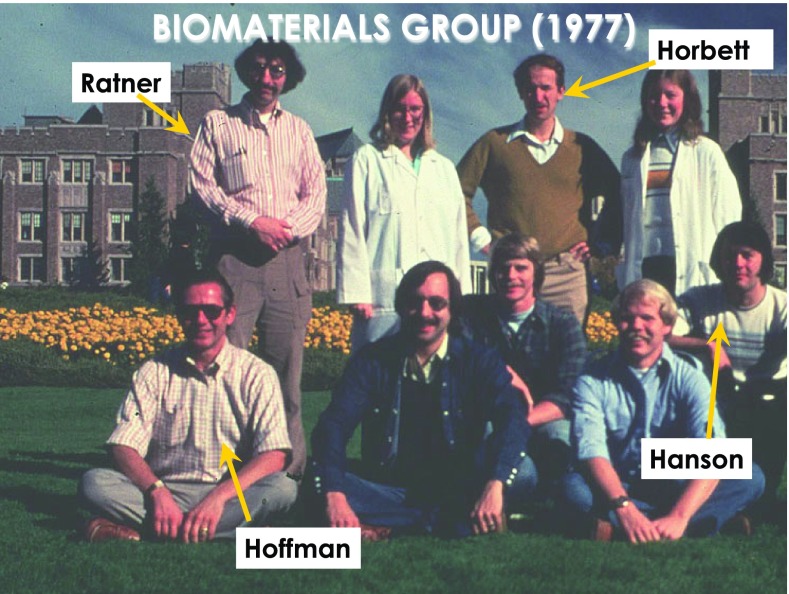
The UW Biomaterials Group in the late 1970s.

**Fig. 2.**
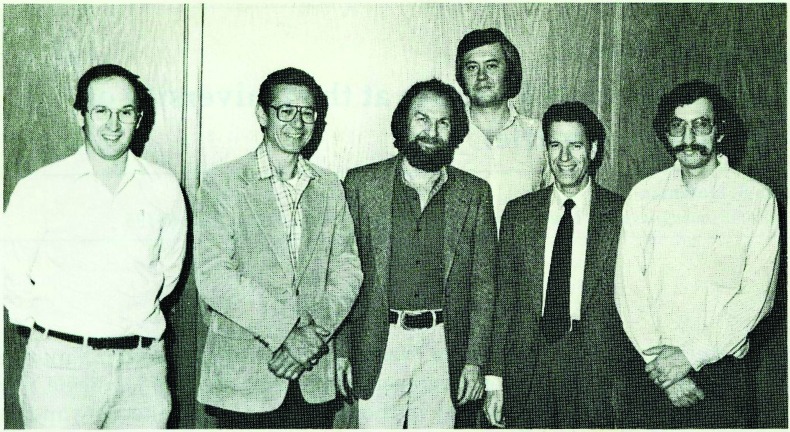
The UW Biomaterials faculty in the early 1980s. From left to right: Profs. Thomas A. Horbett, Allan S.Hoffman, Larry O. Reynolds, Stephen R. Hanson, Laurence A. Harker, and Buddy D. Ratner.

**Fig. 3.**
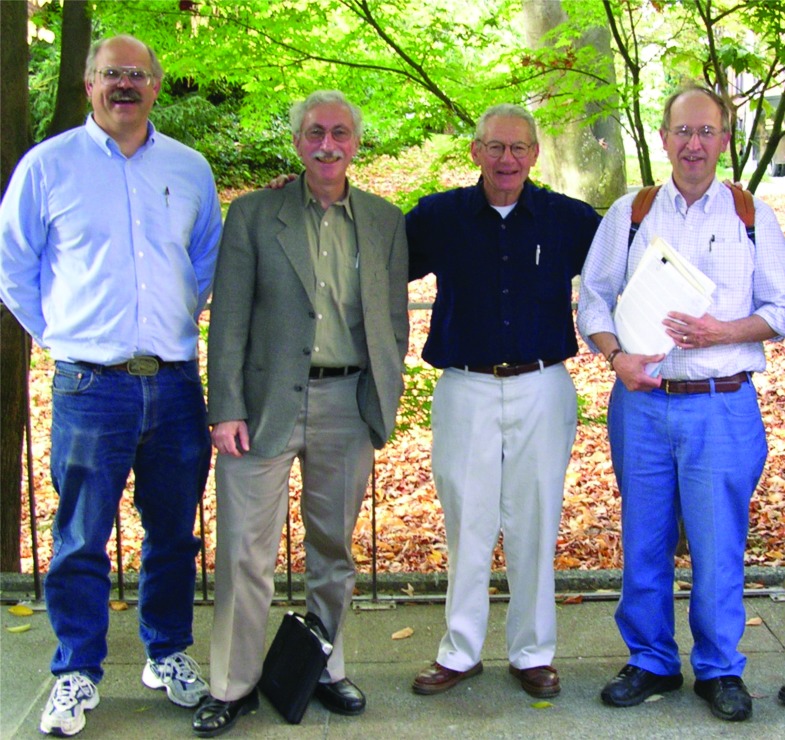
The UW Biomaterials Family Tree (photo taken in 2002). Left to Right: Professors David G. Castner, Buddy D. Ratner, Allan S. Hoffman, and Thomas A. Horbett.

In the late 1970s and early 1980s, our UW biomaterials research group now included myself, plus Tom and Buddy. Figure 1 shows a photograph of our group around the end of the 1970s. We became leaders in studies on the interactions of blood with synthetic acrylic-based hydrogels. We had a team that designed and created novel biomaterials with acrylic surface compositions that had varying hydrophilic/hydrophobic ratios. We measured protein adsorption and platelet adhesion on the surfaces and correlated those data with the varying surface compositions. Later on in the 1980s, we were fortunate to add Larry Reynolds, a physicist who used ultrasound to measure platelet aggregation caused by the contact with our biomaterial surface. In addition, we added Steve Hanson, a Ph.D. student working with Laurie Harker, MD, a hematologist, on platelet aggregation caused by contact with our surfaces. We circulated platelet rich plasma in a shunt and measured scattering of ultrasound in the shunt caused by the formation of platelet aggregates. We correlated those data with surface water content and composition. Some of our conclusions were controversial because we found that the higher the water content in the grafted surface compositions, the more damaging the surfaces were to blood platelets. Figure 2 shows our full biomaterials research group in the 1980s, and Fig. 3 shows a more recent photograph taken in 2002 with Dave Castner, as a fourth key member of our Biomaterials Group.

These efforts stimulated Buddy to conceive the idea of a National Surface Analysis Center to provide modern surface instrumentation to the biomaterials community. He studied and learned all he could about x-ray photoelectron spectroscopy (XPS, also known as electron spectroscopy for chemical analysis or ESCA). He went to Salt Lake City to work with Joe Andrade and his XPS facility. He also teamed up with Dave Briggs to learn all he could about XPS and secondary ion mass spectrometry, plus made many trips to other places such as Hewlett-Packard to work with their XPS equipment.

In the 1980s, Buddy and Tom also each began to pursue their independent careers. In one of their early experiments together, they found that when they implanted gold and polyethylene materials into animals, the healing response was essentially indistinguishable between the two, which they described as the “classic foreign body reaction” when the body encapsulated the implants in collagen scar tissue. This led them, in 1984, to write a major grant application to the NIH to create the National ESCA and Surface Analysis Center for Biomedical Problems (NESAC/BIO). This center has become a world-renowned site for the development of techniques studying biologic interactions of proteins and cells with solid surfaces. It was funded for 14 years under Buddy's direction, and now it continues under the Direction of Professor Dave Castner, Buddy's former postdoc. With the advent of other techniques such as scanning probe microscopy and sum frequency generation vibrational spectroscopy, Buddy and Dave added these methods to their arsenal of surface tools.

In 1983, Buddy and Tom conceived of and published data on a reversible, *p*H-responsive controlled release system, a glucose-sensitive hydrogel that released insulin by acid-induced swelling of the hydrogel membrane. These papers were the first ones published in what is now known as the field of *stimuli-controlled drug delivery*, a field that has grown enormously since their hugely influential first paper was published.

In 1995, Buddy and Tom sent a proposal to the NSF for an engineering research center that would take new developments in biology and apply them to enhance or improve the healing and integration of the biomaterials that were being widely used in medical devices at that time. The NSF liked this idea and invested 40 million dollars into the UW for this program, which Buddy called “UWEB.” UWEB led to the identification of a few special classes of materials that, instead of being walled-off, actually integrated and healed into the body. They realized that they could use these new materials for electrodes in sensing or stimulating devices, and could keep those devices “going” *in vivo.* In addition, if they were used in drug delivery devices, they could prevent the encapsulation that would otherwise gradually block the delivery of the drug molecules. There are many other examples of where such integrated healing provides a very different outcome for a medical device. It permitted many new biomaterials and devices that were not possible before. Buddy has said that the funding of this broad-ranging program is “*one of the hallmark achievements in my career.*” It provided the funding of a team of approximately 20 faculty members to collaborate together and focus very tightly on an important medical problem. He directed UWEB for 12 years under NSF funding and he continues to direct it now as an independent program.

Out of these collaborations came successful materials that have been translated to clinical use by different companies. One of the most significant of Buddy's inventions within UWEB was the creation of a novel microporous biomaterial that he called STAR^®^ (“Sphere Templated Angiogenic Regeneration”). It was developed together with one of his students, Andrew Marshall, who is now a Research Professor in Bioengineering at the University of Washington. STAR revolutionized healing because it induced tissue ingrowth in implants. The STAR porosity was optimized to maximize cell ingrowth and tissue regeneration. Buddy and Andy have together developed a wide range of implant applications based on these novel microporous biomaterials.

This development of microporous scaffold biomaterials has been licensed by UW to Healionics, a Seattle spin-off company. Healionics' first product is now on the market (a veterinary glaucoma shunt). These microporous materials are also being used to study the healing process *in vivo* as living cells invade the pores when the STAR material is implanted within bone, skin, heart, vagina, and sclera. The vascularized, reconstructive, and regenerated tissue achieved with the STAR material is based on simple, low cost synthetic polymers, and it could revolutionize many medical implants in the future.

In another example of translation of his inventions, he has developed drug delivery from an intraocular lens (IOL). The drug-loaded IOL is now licensed to Inson Medical. It could have a huge impact in permitting cataract surgery in third world countries by releasing antibiotics. At this time, cataracts are the world's leading cause of blindness. In the western world, the IOL surgery is routinely performed, but in India, Africa, and China, appropriate operating theaters are not available. This drug delivery system innovation directly addresses this problem.

## Current

Buddy is currently organizing and attracting funding for his new Center for Dialysis Innovation. Buddy will bring into his new endeavor many of the latest advances made in the fields of molecular biology, gene design, disease treatments, protein structure and functioning, and biomaterials, just to name a few of the scientific areas that he will include in this new Center.

In summary of Buddy's qualities and accomplishments, he combines great vision with an exciting and creative imagination, and I believe this unique and powerful combination is why he has emerged as such an outstanding innovator in applications of biomaterials as implants and medical devices. Buddy has often and accurately been cited as “the pioneer in bringing modern surface science to biology and medicine.” He was elected to the National Academy of Engineering in 2002, a major recognition of his huge contributions to the fields of bioengineering and biomaterials. Buddy is also a great lecturer and he is constantly in demand as a Plenary speaker at meetings around the world. As such, he has been a great inspiration and mentor to many, many academic, governmental, and industrial researchers and their students and collaborators around the world. I think it is fair to say that Buddy is *the most renowned and respected professor in the biomaterials field around the world today.*

Buddy Ratner's top 30 publications over the past 45 plus years at the University of Washington:
